# Effect of handoff skills training for students during the medicine clerkship: a quasi-randomized Study

**DOI:** 10.1007/s10459-015-9621-1

**Published:** 2015-07-15

**Authors:** Juan A. Reyes, Larrie Greenberg, Richard Amdur, James Gehring, Linda G. Lesky

**Affiliations:** Division of Hospital Medicine, Department of Medicine, The George Washington University School of Medicine and Health Sciences, 900 23rd St, NW, Washington, DC 20037 USA; The Clinical Learning and Simulation Skills Center, Office of Medical Education, The George Washington University School of Medicine and Health Sciences, Washington, DC 20037 USA; The George Washington University Medical Faculty Associates Biostatistics Core, Washington, DC 20037 USA

**Keywords:** Handoff, Handover, Medical education-clinical skills training, Medical education-undergraduate, Signout, Transfer of care

## Abstract

Continuity is critical for safe patient care and its absence is associated with adverse outcomes. Continuity requires handoffs between physicians, but most published studies of educational interventions to improve handoffs have focused primarily on residents, despite interns expected to being proficient. The AAMC core entrustable activities for graduating medical students includes handoffs as a milestone, but no controlled studies with students have assessed the impact of training in handoff skills. The purpose of this study was to assess the impact of an educational intervention to improve third-year medical student handoff skills, the durability of learned skills into the fourth year, and the transfer of skills from the simulated setting to the clinical environment. Trained evaluators used standardized patient cases and an observation tool to assess verbal handoff skills immediately post intervention and during the student’s fourth-year acting internship. Students were also observed doing real time sign-outs during their acting internship. Evaluators assessed untrained control students using a standardized case and performing a real-time sign-out. Intervention students mean score demonstrated improvement in handoff skills immediately after the workshop (2.6–3.8; *p* < 0.0001) that persisted into their fourth year acting internship when compared to baseline performance (3.9–3.5; *p* = 0.06) and to untrained control students (3.5 vs. 2.5; *p* < 0.001, *d* = 1.2). Intervention students evaluated in the clinical setting also scored higher than control students when assessed doing real-time handoffs (3.8 vs. 3.3; *p* = 0.032, *d* = 0.71). These findings should be useful to others considering introducing handoff teaching in the undergraduate medical curriculum in preparation for post-graduate medical training.

*Trial Registration Number* NCT02217241.

## Introduction

In an effort to reduce errors associated with sleep deprivation, the Accreditation Council for Graduate Medical Education (ACGME) introduced resident duty hour regulations (Accreditation Council for Graduate Medical Education 2013). Cross-coverage and night float system, implemented to ensure that residents do not exceed duty hour limits, rely on patient handoffs to transfer the care of patients from one physician to another (Horwitz et al. 2006). A consequence of reduced duty hours has been greater discontinuity in patient care and a significant increase in the number of handoffs of patients to other physicians (Vidyarthi et al. 2006).

Discontinuity in patient care is associated with increased in-hospital complications (Laine et al. 1993), diagnostic test delays (Laine et al. 1993), preventable adverse events (Petersen et al. 1994), and likely increased cost due to unnecessary tests being ordered by residents not familiar with the patient (Lofgren et al. 1990). There is also evidence of negative consequences due to poor communication and information loss associated with inadequate handoffs (Sutcliffe et al. 2004; Greenberg et al. 2007; Gandhi et al. 2006; Kachalia et al. 2007).

In response to these concerns, numerous national organizations, including the National Quality Forum (2010) and the Joint Commission (2007), have called for increased education about and more standardized approaches to patient care handoffs. In addition, the AAMC Core Entrustable Professional Activities for graduating medical students includes handoffs as a milestone (Aschenbrener et al. 2014).

Since first-year residents are expected to hand off patients effectively beginning their first day of residency, training in these skills is appropriate for undergraduate medical education. A survey of rising fourth-year students at two medical schools revealed that medical students are already participating in the handoff process (Arora et al. 2013). Focus groups conducted at six medical schools confirmed student participation in patient handoffs, often times with little oversight, and exposed the need for formal training (O’Toole et al. 2013).

Although numerous articles on effective handoffs have been published (Solet et al. 2005; Gordon and Findley 2011; Riesenberg et al. 2009), few studies have addressed teaching or evaluating handoff skills at the medical student level. In addition, none of these studies assessed the retention of learned skills over time or the transfer of skills from the simulated setting to the clinical environment.

In this study, third-year internal medicine clerks received training in handoff skills and were followed into their fourth year to assess the durability of the training and the transfer of skills into the clinical setting.

## Methods

### Design

**Fig. 1:**
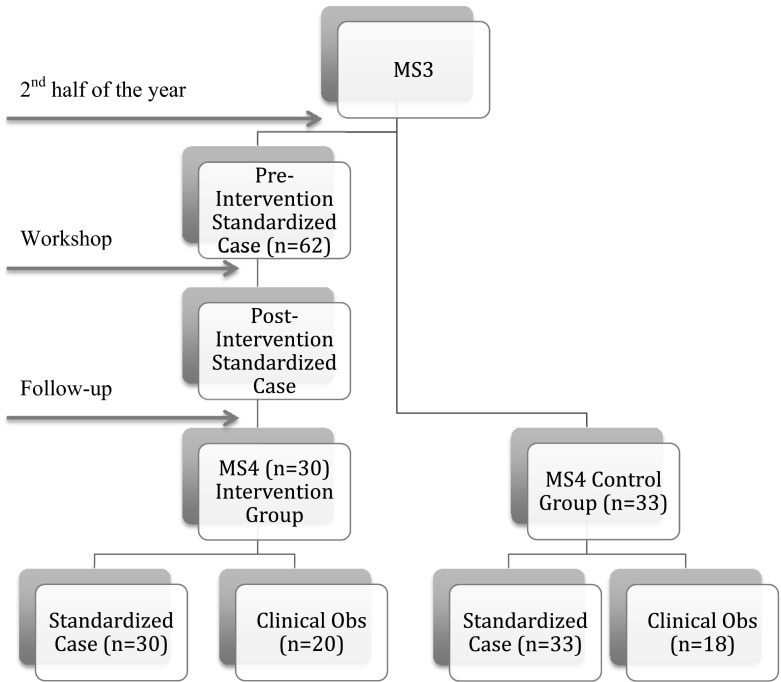
Study design

Third-year medical students from The George Washington University spend 1 month of their internal medicine clerkship at the George Washington University (GWU) hospital. While students can specify a preference for the timing of a particular clerkship, final placement is determined by a lottery-based allocation system. Following approval by GWU institutional review board, we conducted an intervention study of third-year internal medicine clerks beginning in the second half of the academic year (January 2012–June 2012), thus quasi-randomizing the third-year class into intervention and control groups. Students rotating on internal medicine during the first half of the academic year did not receive the intervention. Approximately 40 % of these students complete their acting internship (AI) at the university hospital and served as controls during the follow-up period (Fig. 1).

**Fig. 2:**
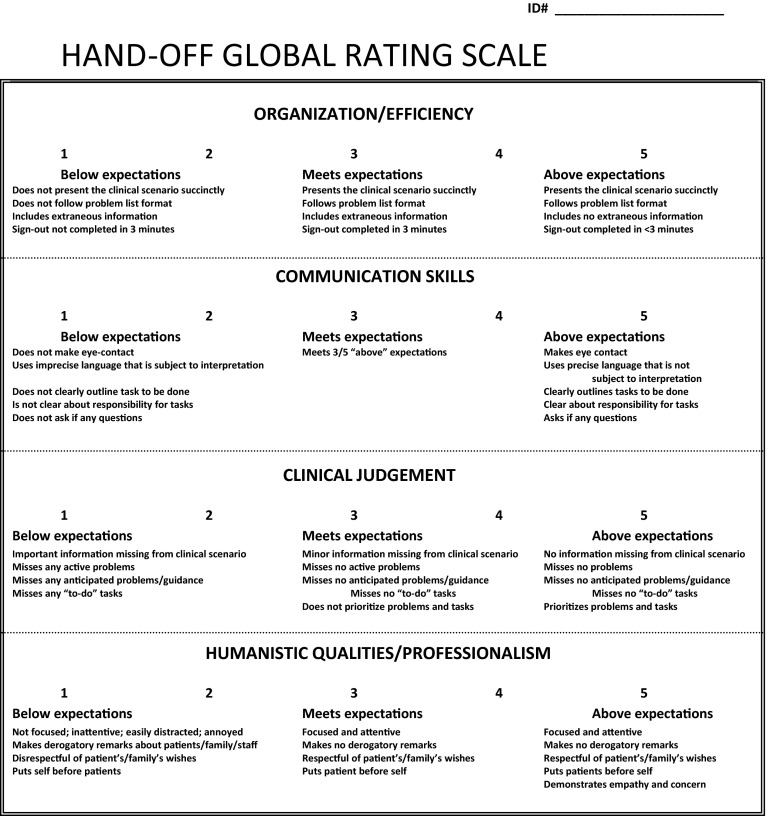
Handoff Global Rating Scale

Three standardized cases were developed to evaluate a student’s verbal handoff skills. The cases were constructed specifically to avoid examining a student’s medical knowledge, but rather to assess the student’s ability to extract the relevant information for a complete and concise handoff. The first case served as a pre-intervention baseline assessment of third-year medicine clerks. The second case evaluated the third-year student’s skills immediately following the intervention, and the final case was used to assess handoff skills during the student’s 4th year AI. We designed a handoff assessment tool (Fig. 2), utilizing the four performance domains outlined by Farnan et al. (2009). Each domain was described along a 5-point rating scale with specific behavioral anchors for each. The domains focused on organization and efficiency, communication skills, clinical judgment, and humanistic qualities/professionalism. The assessment tool was revised in response to feedback from faculty hospitalists who reviewed the domains and behavioral anchors for content validity. Four volunteer students assisted in training six faculty observers to use the Handoff Global Rating Scale in the simulated setting. Utilizing the three standardized cases, each student delivered an end-of shift verbal handoff. Faculty trainees were provided with written copies of the cases and assessed each student at the end of the presentation. Using an iterative process the faculty worked towards agreement on the rating for each domain of each student’s presentation of each of the three standardized cases. The students presented in a different order for each of the three cases. By the fourth presentation of each case, the trained faculty had achieved an interrater reliability of kappa = 0.89. Two additional faculty observers, blinded to student intervention status, were trained to use the rating scale during real time sign-outs, and achieved an interrater reliability of kappa = 0.78 for 4 real-time patient handoffs observed in the clinical setting. The trained observers, faculty physicians from the Divisions of Hospital Medicine and General Internal Medicine, were carefully selected for a particular session to ensure that they had no responsibilities for teaching or evaluating students they were observing.

### Intervention

**Fig. 3:**
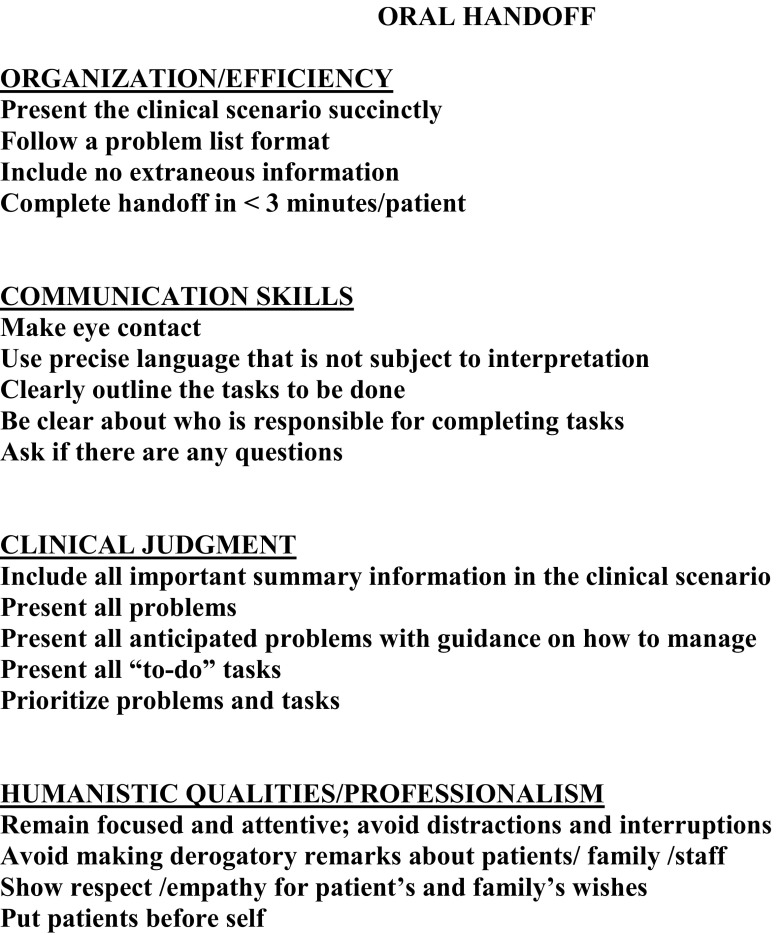
Pocket card

Third-year students in the intervention group participated in a 1-h interactive, small-group workshop facilitated by a study investigator. The workshop initially focused on the importance of specific handoff skills to patient safety. Utilizing an example of a poor sign-out, students worked together to identify the critical elements of an effective handoff. They were provided with a standardized format for both an oral and written handoff and received a pocket card (Fig. 3) highlighting the required elements. Students practiced handing off a case and received feedback prior to being evaluated with the post-intervention standardized case. Individualized feedback was given to each student after the post-intervention handoff. At the beginning of the student’s fourth-year AI, each student, including those in the control group, received the pocket card as part of their orientation materials. No further instruction was provided except that they could use the card for guidance when handing off patients.

Supervising residents and attendings on the wards were unaware of their students’ involvement in the study, and during the study period, there was no formal handoff training for residents or interns.

### Outcome measures

To assess the effectiveness of the workshop, third-year students in the intervention group were evaluated using standardized cases 1 week before and immediately after the workshop. The trained observers were blinded to the timing of the intervention. The durability of the training was assessed during the subsequent academic year by evaluating intervention and control group fourth-year AI students using a third standardized case. For this case, the trained faculty evaluators were blinded to the student’s intervention status. The transfer of handoff skills into the clinical setting was assessed by observing intervention and control group fourth-year AI students, conduct real-time handoffs. Again, trained faculty observers were blinded to the student’s intervention status. Third-year intervention students completed a retrospective pre/post self-assessment of performance and perceived effectiveness (Skeff et al. 1992) of the educational intervention at the conclusion of the post-intervention standardized case. They were also asked about their prior experience and training in patient handoffs.

### Statistical analysis

SAS version 9.2 was used for all analyses with *p* < 0.05 considered significant. Data distributions were examined for normality and outliers. To examine changes in the mean scores of the third year students in the intervention group across time periods (pre-intervention versus post-intervention third-year students, third-year pre-intervention versus fourth-year intervention students, and third-year post-intervention versus fourth-year intervention students), 2-tailed paired t-tests were used. For each pair of time points, Pearson correlation (r) was used to examine whether students with the highest initial scores also had the highest later scores. Differences between the fourth-year students intervention and control group scores in the standardized case and real-time clinical handoffs were tested using 2-tailed independent groups *t* test. Cohen’s *d* was used to examine effect size between students in the intervention versus control groups, for both the standardized patient case as well as real-time observed handoffs.

## Results

Sixty-five third-year medical students rotating on the inpatient medicine service from January 2012 to June 2012 were eligible for the pre-intervention group and 62 (95 %) agreed to participate. Thirty of these intervention students (48 %) completed their fourth-year AI at the university hospital and participated in the standardized case evaluation. Thirty-three students completing their third-year medicine clerkship during the first half of the academic year and did not undergo the workshop training served as controls during their fourth-year AI and participated in the standardized case evaluation. Twenty-fourth-year students (32 %) from the intervention group (n = 62) and eighteen control students were observed doing a real time sign-out in the clinical setting (Fig. 1).

**Fig. 4:**
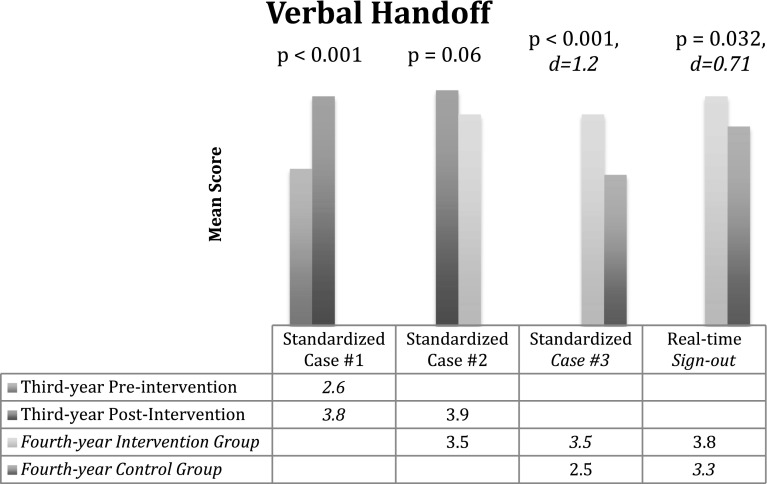
Study results—Verbal Handoff Mean Average Scores of intervention group for standardized patient case (SP) during third-year pre-intervention, third-year post-intervention and follow-up into their fourth-year acting internship. Verbal Handoff Mean Average Scores of intervention and control group during their fourth-year acting internship for a standardized patient case and real-time signout

Following the workshop intervention, 56 of 62 third-year students (90 %) demonstrated improvement when compared to their pre-intervention mean score. On average, students increased their mean score by 30 % (2.6–3.8; *p* < 0.001). Among third-year intervention students followed into the fourth year (n = 30), after an average of 9 months from the initial workshop intervention, there was a 10 % non-statistically significant decrease in mean score (3.9–3.5; *p* = 0.06) when compared to scores assessed immediately after the workshop. When compared to their pre-intervention baseline, 63 % of 4th year intervention students maintained an improvement in mean score (2.9 vs. 3.5; *p* = 0.015, r = 0.03). When compared to fourth-year students in the control group (n = 33), fourth-year intervention students achieved higher mean scores (3.5 vs. 2.5; *p* < 0.001, *d* = 1.2) for the standardized patient case. Intervention students (n = 20) scored higher than students in the control group (n = 18) during their fourth-year AI when assessed doing real-time sign-outs (3.8 vs. 3.3; p = 0.032, *d* = 0.71) (Fig. 4).

Retrospective self-assessment revealed that 72 % of third-year intervention students felt unprepared or somewhat unprepared to perform an effective handoff prior to completing the educational workshop. Ninety-seven percent reported no formal training apart from the educational workshop. At the conclusion of the intervention 75 % of third-year students felt well or very well prepared to perform an effective handoff. Eighty-six percent of students reported the workshop to be effective or very effective.

## Discussion

This study is a quasi-randomized controlled trial conducted among third-year medical students designed to evaluate the immediate impact of an educational intervention, the durability of learned skills into their fourth year Acting Internship, and the transfer of skills from the simulated to the clinical setting. Third-year students participating in a skill-focused, 1-h small-group workshop on handoff skills demonstrated improvement in the performance of a verbal handoff immediately after the intervention and 9 months later when compared to untrained control students. Although intervention students showed a predictable decrement in performance in the simulated setting after an average follow-up of 9 months into their fourth-year AI, they continued to demonstrate improved performance compared to their baseline skills and to untrained control students. When conducting real-time handoffs in the clinical setting, fourth-year students in the intervention group performed better than untrained controls. In addition, the handoff assessment tool was demonstrated to distinguish between trained and untrained students and to detect decrements in performance over time. Finally, in a retrospective self-assessment, the majority of students felt the educational intervention to be effective and felt better prepared to perform a handoff at its conclusion

There may be several explanations grounded in adult learning theory that could help explain student’s improved skills immediately after the intervention and the performance transfer into their fourth-year AI. The intervention was designed to ensure that students were actively involved in the learning process as opposed to passive observers. They also are more interested in immediate, problem-centered approaches versus subject centered ones. Since students participate in handoffs during their clinical experiences, they realized these skills would be immediately useful for them in their clinical education. Their ongoing patient care experiences in the clerkship years and during their critical fourth year when they assume more responsibility were also motivators for learning and performing. Lastly, they had a readiness to learn and use new knowledge and skills that would enhance their contribution to real patient care.

Most research on the handoff process has focused at the resident and attending level (Gordon and Findley 2011; Riesenberg et al. 2009; Farnan et al. 2009; Curtis et al. 2013; Horwitz et al. 2007; Chu et al. 2009; Foster and Manser 2012). These studies have been predominantly observational and assessed self-perceived performance and comfort conducting a patient handoff. Only a limited number of curricular interventions have been evaluated experimentally and few instruments validated to evaluate handoff skills (Horwitz et al. 2013; Pezzolesi et al. 2013). Gakhar and Spencer (2010) reported improvement in written and verbal handoff skills 8 weeks after an educational intervention, but did not include a control group. Starmer et al. (2013) demonstrated a decrease in medical errors and adverse events after the introduction of a resident handoff bundle that included standardized handoff training, a verbal mnemonic, and a new team handoff structure. The study did not include a control group and increased resident experience likely contributed to the improvement seen over time. Airan-Javia et al. (2012) demonstrated that a handoff education session improved the quality of verbal handoffs skills amongst interns. However, its effectiveness was measured over a period of only 2 weeks.

There are fewer studies addressing handoff training for students, despite the fact that students report participating in handoffs (Arora et al. 2013; O’Toole et al. 2013) and entering housestaff are expected to be proficient (Association of Program Directors in Internal Medicine 2010). Klamen et al. (2009) reported improvement in second-year medical student handoff skills following an educational intervention, but did not include a control group. Darbyshire et al. (2013) demonstrated improved knowledge and satisfaction following a 1-h educational session for senior students, but the study was not designed to evaluate handoff skills. Chu et al. (2010) described a Handoff Selective for rising fourth-year medical students and reported improved self-perceived understanding and performance of handoffs, but no objective measures of performance.

Our study has several limitations. First, it was conducted at a single institution during an internal medicine clerkship and may not be generalizable to other institutions or disciplines. Second, student assignment to intervention and control groups may not have been entirely random. Students can specify a preference for the timing of a clerkship. However, it is not clear how a student’s preference to complete an internal medicine rotation during the second half of the academic year would bias the results. By carrying out the intervention among more seasoned third-year students we potentially limited the impact of the intervention, particularly if they had received handoff training during other clerkships. Arguably, it did shorten the time to follow-up assessment in the 4th year. Still, the average follow-up time was 9 months. The use of only one case for each assessment is an additional limitation. However, this is more a reflection of time limitations of both students and faculty in being able to carry out this project longitudinally during the clinical years. We would argue, however, that despite this we were able to demonstrate a significant change in absolute scores between intervention and control group associated with an effect size of modest and high practical significance for real-time signout and standardized patient case respectively. Finally, the study was conducted among medical students and, as such, was not designed to assess the impact on patient outcomes of an intervention to improve handoff skills. Despite these limitations, we believe this study has several major strengths, including the quasi-experimental design, the duration of student follow-up, the development of an effective 1-h interactive workshop and the development of an evaluation tool to assess students’ handoff skills in both the simulated and clinical setting. We believe the results from this study can be generalized to other institutions to help prepare students for their PGY-1 year.

## Conclusions

Our study resulted in a model for training students in handoff competency skills that is concise, effective and durable. In addition, we have provided validity evidence for an assessment tool of handoff skills. We demonstrated it to be accurate and reliable for discriminating levels of performance among students in both the simulated and clinical setting. With training, faculty utilized the tool with a high degree of inter-rater reliability. Future studies are needed to further validate the assessment tool in other clinical disciplines and with learners at different levels of training.
